# The diversity of skills that are needed in different environments

**DOI:** 10.3389/fsurg.2023.1240154

**Published:** 2023-09-20

**Authors:** A. David Mendelow

**Affiliations:** Newcastle University, England, United Kingdom

**Keywords:** neurosurgery, image guidance neurosurgery, stereoradiosurgery, spinal neurosurgery, inversion therapy, deep brain stimulation, neurotrauma, neuronavigation

## 1. Neurotrauma

Traumatic Brain Injury (TBI) is often the raison d'etre for the existence of neurosurgeons, particularly in LICs but it remains the main reason for maintaining neurosurgical services throughout the world. Rapid access to imaging has made it possible to minimise delays in the evacuation of hematomas: predominantly with the CT scanner. Most trainees gain their initial experience in neurotrauma in the form of craniotomies, craniectomies, burrholes and ventricular catheterization. The transmission of images with PACS systems and even mobile phones has greatly simplified the consultation process. Where resources permit in HICs, the large decompressive craniectomy has ensured the survival of many patients with severe head injury (1, 2). Large International databases of TBI have been set up and are available for auditing neurotrauma services (3–5). The recent publication of ICD11 has made it possible for all neurotrauma coding hospitals to insert the Glasgow Coma Scale (GCS) and pupil reactions directly after the Head Injury Code (6), making it possible to compare the patterns and Mortality of TBI patients within and between all LIC, MIC and HIC countries, using post-coordination prognostic clinical indicators for the first time. The incidence of trauma generally decreased during the COVID-19 Pandemic, but facilities remained open for TBI care (7).

## 2. Image guidance with stereotactic methods

The development of Image Guided surgery using pre-operative or intra-operative CT, MRI or magnetism can be deployed with BrainLab or Medtronic's Stealth system or Philips' Zeniton or Azuron or Stryker's Q Guidance system amongst others, but all require specific training and equipment. The most recent development is Frameless stereotactic surgery where fiducial markers are fixed in place and instruments are reformatted and tracked in real time in 3D in relation to the pre-operative or even intra-operative scans. By contrast, intra-operative angiography or intra-operative ultrasound scanning can easily be learned and used anywhere in the world at modest cost and are suitable for LICs. Stereotactic brain surgery has existed for more than a century now, having been first used experimentally in 1908 by Sir Victor Horsley at UCH in London and later used in humans in the 1940s by Spiegel and Wycis from the USA and Leksell from Sweden. The stereotactic 3D metal frame he developed still bears his name as does the Radiosurgery system he pioneered in the form of the Gamma Knife. The radioactive cobalt system he made is still in use today in a much more accurate form by Elekta. The concept of focussed radiosurgery has evolved so that robotic linear accelerators could move around the patient (Cyberknife's first model from 1987) and later their M6 and S7 models became faster and more accurate. They went on to incorporate BrainLab elements into the latest Cyberknife under the name “Accuray”. Such advanced technological developments used multileaf collimator technology to deliver precisely focussed stereo radiotherapy to small targets deep within the brain with minimal radiation to surrounding heathy tissues. Linac, Tomotherapy, Accuray and BrainLab are all examples of modern but expensive brands used predominantly in HICs where it is predicted that, with Proton Beam Therapy, there will be massive expansion of these technological advances over the next decade (8).

## 3. Different nursing environments

There has been a general recognition that good critical care environments improve outcomes in neurological patients, particularly with stroke and neuro trauma (9). Although there are differences in stroke care organisation between MIC and HIC, most of these hospitals had dedicated stroke care units, but HIC hospitals had greater access to rehabilitation and a higher proportion of patients receiving intravenous thrombolysis compared to MIC hospitals (10). These facilities were often overwhelmed during the COVID-19 Pandemic but hospital avoidance may account for some of the increased mortality (11). One of the technological developments that has improved care has been the bedside monitor. In addition to the standard Cardiorespiratory modalities that are in frequent use, Intracranial Pressure, Microdialysis, Electrophysiological and Transcranial Doppler monitors are now easier to use and more accurate, providing continuous parameters rather than just “snapshot” events. Some of these new skin-patch and percutaneous monitoring techniques have become “wearable” so that patients can monitor their ECG, glucose, hormonal changes and other physiological variables at home, thus creating the concept of a “virtual ward” outside the hospital. At the other end of the spectrum is the CT, PET and MRI imaging facility attached directly to the HIC Intensive care unit: another example of the extremes of diversity that exists in modern critical care.

## 4. Spinal surgery

**Figure 1:**
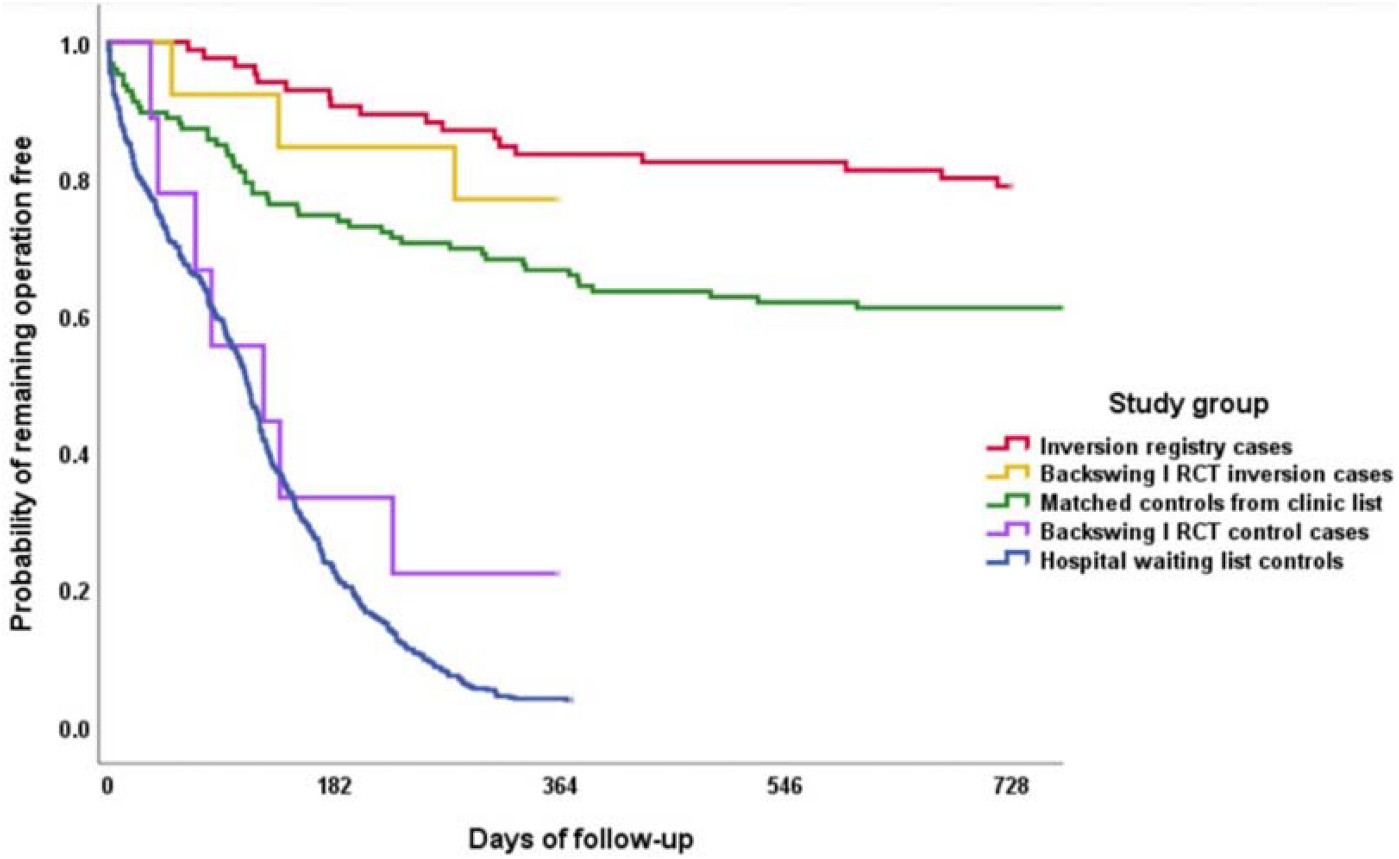
From Mendelow et al. 2021 (15). Probability of remaining surgery free in the inversion registry (red) vs. Matched controls (green) from a UK NHS neurosurgery clinic list (Logrank test *p* < 0.001). Backswing I Inversion cases (yellow) and controls (purple) were from the previous Prospective Randomized Controlled Trial (14). Hospital discectomy NHS waiting list controls (blue) were from the Neurosurgery lumbar spine operative waiting list in 2014, followed through to 2015. Reproduced with permission of the Society of Physical Therapy Science from Mendelow A D et al. Lumbar disc disease: the effect of inversion on clinical symptoms and a comparison of the rate of surgery after inversion therapy with the rate of surgery in neurosurgery controls. *J Phys Ther Sci* 33: 801–808, 2021.

The greatest contrast in neurosurgical case-mix between LICs hospitals and those in other countries is with Spinal surgery. Very few degenerative spinal conditions are treated surgically in LIC hospitals, while in Private Hospitals in HIC, Spinal surgery procedures and facilities predominate. This contrast is even greater when spinal instrumentation is considered. In lumbar degenerative disease with stenosis, Clinical trials have failed to show that instrumented fixation is better than decompression alone (12). Clinical trials about any added efficacy (and the costs) of cervical spinal fixation are ongoing (13). An alternative to surgery for degenerative spinal disease (excluding those patients with acute spinal cord or cauda equina compression of course) is disc distraction therapy: in the cervical region over-door pulley traction with a head harness is effective and in the lumbar spine an inversion table can be used to reduce disc protrusions significantly more effectively than discectomies in a neurosurgery department (Figure 1) (14, 15). The inversion table itself could be considered as technological advance, although inversion for spinal disorders was practiced 2,500 years ago by Hippocrates (16). Such inversion therapy is a safe and low cost alternative to lumbar discectomy, provided that patients with Cauda Equina Syndrome (CES) are excluded, because they all (CES patients) need immediate decompressive surgery whether in LICs, MICs or HICs.

## 5. Microsurgery, endoscopy and robotic neurosurgery

The operating microscope revolutionised operative neurosurgery in the 1960s and is today considered an essential piece of equipment in any neurosurgical theatre. Modern technological developments in magnification, lighting and Neuronavigation have created very sophisticated image-assisted robotic instruments with varying degrees of surgical assistance. These include simple robotic arms that hold instruments and or endoscopes to very stable accurate robots that provide steadiness and precision beyond human capacity with brands like the da Vinci Surgical System, the Mazor X Stealth Edition made by Medtronic and the Zeus Robot Surgical System. The robotic concept also applies to endoscopes that can be introduced through various orifices and then made rigid for operative robotic procedures. All the imaging can be relayed onto 2D and 3d screens or VR headpieces with recordings kept on solid state devices. These increasingly expensive advances are likely to remain in the HIC hospitals but Zeiss have produced an accurate versatile operating microscope that provides perfect magnification and lighting for neurosurgery and ophthalmology at a price that suites the LIC hospital budget. Similarly, there is a wide range of operating loupes with lighting and cameras that provide excellent magnification and lighting at low cost. Most surgeons will acquire their own set of loupes, some with built in cameras and recording equipment.

## 6. Imaging, telemedicine and AI

The revolution in brain imaging with CT and MRI scanners has resulted in ubiquitous high-quality neuro diagnosis throughout most of the world today. Other scanners like the PET scan and various combinations with CT and MRI functional imaging have led to a better understanding of neurophysiology and neurobiochemistry. The ability to transfer images, even to the other side of the world, with Telemedicine and the Internet has enabled the interpretation of such images to be relayed back to clinicians instantly. Artificial intelligence programmes like Brainomix (17) make interpretation of complex brain images easy for clinicians faced with decisions about urgent treatment like thrombolysis or thrombectomy. This Brainomix programme is even available as an app for local use. There are many such AI programmes available and AIMultiple lists the top 16 such companies at the time of writing (18). THE World Federation of Neurosurgical Societies (WFNS) has organised a wide variety of surgery simulation courses in LMICs. These include spinal techniques, endoscopy, vascular and trauma demonstrations that avoid the need for expensive and sometimes illegal cadaver dissections. Low cost simulators and live or offline video feeds now allow wide dissemination of practical skills that were not available in the past.

## 7. Functional neurosurgery

There is great media interest in functional neurosurgery with electronic implants and cortical monitoring with computer interfaces. Such ideas are in their infancy and include the development of artificial vision in blind patients and motor coordination that allows paralysed limbs to move with hemiplegia and paraplegia. The problems are the very high cost, which becomes prohibitive in LIC hospitals and the fact that operating on the brain carries the risk of infection and haemorrhage.

Electrical implants and devices have been used for many years with Parkinson's disease (Deep brain Stimulation), where the risks are accepted because the benefits are tremendous for patients (19). So, the technology exists and is used but cost benefit analysis needs to be considered carefully. Similarly, spinal cord stimulation, cranial nerve stimulation and peripheral nerve stimulation have various uses, usually with prior external wires used in the trial pilot phases.

## 8. Research, teaching and training

There is no barrier to Clinical Research, Teaching and Training in any country, these academic pursuits being the prerogative of the individual clinician. A general knowledge of Evidence-Based Medicine (EBM) should be integral and mandatory in any undergraduate and/or postgraduate programme as was originally advocated by David Sackett in 1997 (20). Access to the Cochrane Library with its Extensive Collection of Systematic Reviews is free in many countries of the world and is now available in 17 languages (21). Within Neurosurgery, there are numerous texts of Evidence-Based Medicine (22–25), although there are antagonists as well as protagonists (26). Basic scientific research can also be undertaken in any country, although resource limitations in LICs make it difficult to apply new, complex or expensive methodologies.

Finally exchange schemes between neurosurgical units of LICs, MICs and HICs are very valuable, not only for the individual neurosurgical trainees that spend a year or two in a HIC hospital, but also for the HIC hospital itself where these academic Fellows have helped to advance the scientific field enormously for all of us. Likewise, HIC trainees who spend time in LIC hospitals benefit from the diverse clinical practice that they will encounter in LIC units.
